# Increased expression of Golgi phosphoprotein-3 is associated with tumor aggressiveness and poor prognosis of prostate cancer

**DOI:** 10.1186/1746-1596-7-127

**Published:** 2012-09-24

**Authors:** Xing Hua, Lina Yu, Wenhai Pan, Xiaoxiao Huang, Zexiao Liao, Qi Xian, Li Fang, Hong Shen

**Affiliations:** 1 Department of Pathology, Nanfang Hospital, Southern Medical University, 510515, Tonghe, Guangzhou, People’s Republic of China; 2 Department of Pathology, School of Basic Medical Sciences, Southern Medical University, 510515, Tonghe, Guangzhou, People’s Republic of China; 3 Department of Pathology, the Forth Affiliated Hospital of Jinan University, Guangzhou, China; 4 Department of Pathology, Guangzhou Red Cross Hospital, Guangzhou, China; 5 Department of Urology, the Forth Affiliated Hospital of Jinan University, Guangzhou, China; 6 Department of Urology, Guangzhou Red Cross Hospital, Guangzhou, China

**Keywords:** Prostate cancer, Castration resistant, Golgi phosphoprotein-3, Prognosis, Tissue microarrays

## Abstract

**Background:**

To investigate the expression of Golgi phosphoprotein-3 (GOLPH3) in prostate cancer and determine its prognostic value.

**Methods:**

Immunohistochemical staining for GOLPH3 was performed on tissue microarrays of 342 prostate patients. The correlation between GOLPH3 expression with its clinicopathologic factors was also analyzed in order to determine its prognostic significance.

**Results:**

GOLPH3 expression of normal prostate tissues, benign prostate hyperplasia, high-grade prostatic intraepithelial neoplasia, and hormone-dependent prostate cancer (HDPC) did not show any statistically significant difference. In contrast, statistically significant difference was reported in moderate/intense GOLPH3 expression in cases diagnosed with HDPC and castration resistant prostate cancer (CRPC) (*P* < 0.0005). Moderate /intense expression of GOLPH3 was associated with androgen independence (*P* = 0.012), higher Gleason score (*P* = 0.017), bone metastasis (*P* = 0.024), higher baseline prostate-specific antigen (PSA) (*P* = 0.038), and higher PSA nadir (*P* = 0.032). A significantly negative correlation was found between moderate/intense GOLPH3 expression and disease-free survival (DFS) (HR = 0.28, *P* = 0.012) and overall survival (OS) (HR = 0.42, *P* = 0.027). Univariated analysis indicated that moderate/intense GOLPH3 expression created a significantly prognostic impact in patients with CRPC. On the other hand, multivariate analysis indicated that GOLPH3 was a significantly independent prognostic factor of DFS (*P* = 0.027) in all prostate cancer patients.

**Conclusions:**

In this study, it was discovered that the overexpression of GOLPH3 is associated with the transition of prostate cancer from hormone sensitive phase to hormone refractory phase. GOLPH3 might be an important prognostic factor of DFS and OS in patients with prostate cancer. In totality, GOLPH3 could be used as a novel candidate in devising a more effective therapeutic strategy to tackle CRPC.

**Virtual slides:**

The virtual slide(s) for this article can be found here: http://www.diagnosticpathology.diagnomx.eu/vs/1452541171722856.

## Background

Prostate cancer is a major public health problem. According to reported estimates, prostate cancer is regarded as the second most common malignancy among men residing in the European Union and North America. In recent years, the morbidity rate of prostate cancer has been increasing steadily in China. For example, the annual morbidity rate of prostate cancer has increased by 14% since 1990. In contrast, the annual morbidity rate was quite stable in the 1970s and 1980s [1].

Most cases of prostate cancer are responsive to androgen ablation therapy in the initial stages. However, many tumors eventually become androgen-refractory. Thus, these tumors become resistant to hormonal therapy with the passage of time. Eventually, metastatic phenotypes proliferate in patients suffering from prostate cancer [2]. We have not been successful in devising an effective therapeutic approach to tackle cases of castration resistant prostate cancer (CRPC). In fact, few biomarkers are capable of reasonably distinguishing aggressive and non-aggressive tumors after diagnosis. In other words, biomarkers with greater sensitivity and specificity can provide evidences for the diagnosis and prognosis of CRPC [3,4].

Golgi phosphoprotein-3 (GOLPH3) has several alternative names, such as GPP34, GMx33, MIDAS, and yeast Vps74p. It is a member of the trans-Golgi matrix family and binds to PtdIns(4)P-rich trans-Golgi membranes and MYO18A. This indicates that a tensile force is required for efficient tubule and vesicle formation [5,6]. Recently, several evidences suggest that GOLPH3 is an oncogene, representing a first-in-class Golgi oncoprotein. GOLPH3, a novel oncogene, is commonly targeted for amplification in human cancer. Note that, an enhanced activation of mTOR signaling represents a molecular basis of GOLPH3’s oncogenic activity [7,8]. However, research studies have seldom studied the correlation between GOLPH3 expression and prognosis of Chinese patients with prostate cancer. In fact, very few studies have explored the transition from hormone-sensitive prostate cancer to CRPC. Taking this fact into consideration, we performed immunohistochemical assay to evaluate the expression of GOLPH3 in definite tissues. We also carried out retrospective follow-up analysis to explore the correlation between GOLPH3 expression and clinicopathologic factors associated with the prognosis of Chinese patients with prostate cancer.

## Materials and methods

We used the surgical prostate cancer database to retrospectively evaluate 342 patients. In the period extending from October 2002 to December 2009, these patients had undergone prostatectomy, transrectal prostate biopsy under ultrasound guidance, or transurethral resection of the prostate. Among the 342 patients, 139 (36.48%) suffered from hormone-dependent prostate cancer (HDPC). On the other hand, 102 (26.77%) patients were diagnosed with CRPC. 61 (16.01%) cases were diagnosed with high-grade prostatic intraepithelial neoplasia (HGPIN). 20 (5.25%) patients were diagnosed with benign prostate hyperplasia (BPH), while 20 (5.25%) cases were normal prostate tissue subjected to pancystectomy. All the specimens were fixed with formalin and embedded in paraffin. All the slides were blindly reviewed by five pathologists, and a consensus diagnosis was reached.

Clinical data, including Gleason score, baseline prostate-specific antigen (PSA), prostate-specific antigen nadir, bone metastasis, and follow-up status, were retrospectively obtained from Hospital Medical Records Room. On the other hand, paraffin-embedded prostate tissues were obtained from the Department of Pathology, the Fourth Affiliated Hospital of Jinan University, Guangzhou, China. The data analysis was approved by our hospital review board.

Tissue microarrays were constructed according to the previously described procedure [9]. Briefly, representative areas of prostate tissue from each of the 342 cases were identified on the corresponding slides stained with hematoxylin and eosin. Tissue cylinders with 1 mm diameter were punched from each donor tissue block so that they could penetrate into a recipient paraffin block. A tissue microarrayer was used in this procedure. The recipient paraffin block was subsequently cut, and the slices were transferred onto coated slides using adhesive tape. Then, the slides were dipped in paraffin to prevent oxidation. With the objective of minimizing tissue loss and problems associated with tumor heterogeneity, every sample was arrayed in triplicates.

To determine the immunohistochemistry of these tissues, tissue microarray sections were stained and GOLPH3 expression was determined according to the procedure described in previous studies [10]. In summary, the primary antibody was raised against GOLPH3 (ProteinTech Group, Inc.; Cat#:19112-1-AP; 1:100 dilution). Staining for GOLPH3 was reckoned as positive provided cytoplasmic staining was observed in more than 10% of definite cells. In cases of positive staining, the intensity of stain were recorded as either weak (1+), moderate (2+), or intense (3+).

Statistical analysis was performed using SPSS16.0 software. Chi-square test was used to investigate the significance of the relationship between GOLPH3 and the individual variables. The relationship between GOLPH3 expression and their clinical outcomes was estimated through both univariate and multivariate analyses. The disease-free survival (DFS) and overall survival (OS) curves were estimated using the Kaplan Meier method, while the differences in the survival curves were compared using the log-rank test. A multivariate analysis was performed using Cox’s regression model. *P* values ≤0.05 were of statistical significance.

## Results

**Figure 1:**
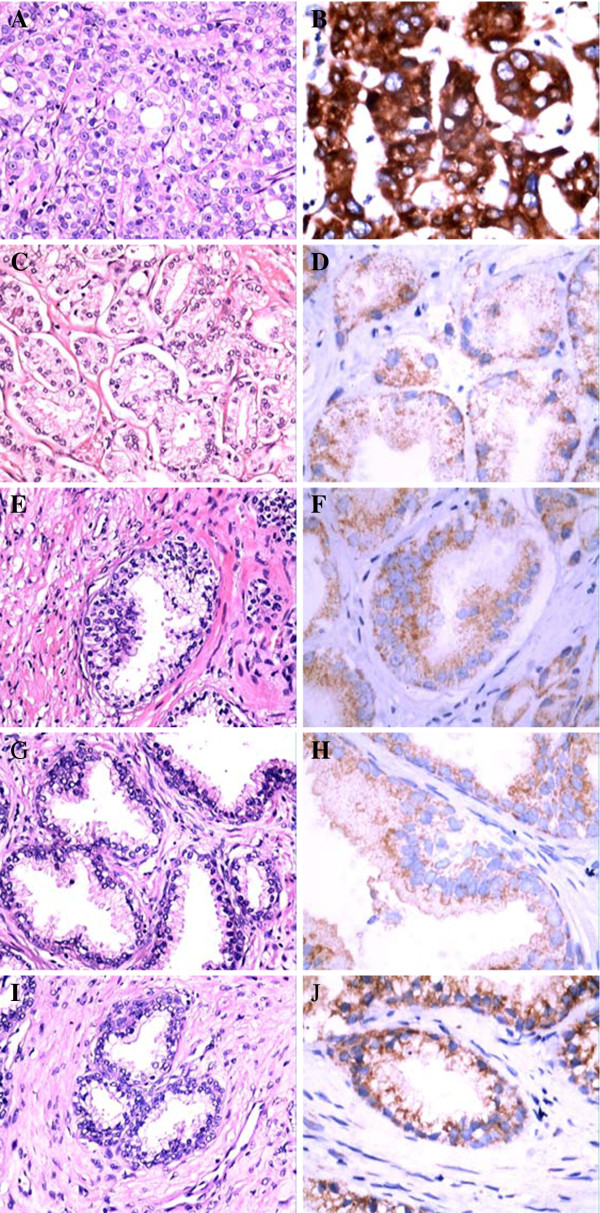
**Representative H&E and immunohistochemical staining results for GOLPH3 (HE x200, IHC x400). A** CRPC, **H&E** staining. **B** CRPC, GOLPH3(+++), **IHC** staining; **C** HDPC, **H&E** staining. **D** HDPC, GOLPH3(+), **IHC** staining; **E** HGPIN, **H&E** staining. **F** HGPIN, GOLPH3(+), **IHC** staining; **G** BPH, **H&E** staining. **H** BPH, GOLPH3(+), **IHC** staining; **I** Normal prostate tissue, **H&E** staining. **J** Normal prostate tissue, GOLPH3(+), **IHC** staining.

In the 342 cases investigated in this study, distinct immunohistochemical staining (negative, 1+, 2+, or 3+) of GOLPH3 was reported (Figure 1). The association of the immunophenotype of GOLPH3 with various clinicopathological parameters has been enlisted in Table 1.

**Table 1:** Clinicopathologic characteristics of patients according to GOLPH3 status

**Characteristics**	**Intensity of GOLPH3 expression**						
	**NNegative**	**%**	**Positive**				
			**Weak (1+)**	**%**	**Intense/moderate(2+/3+)**	**%**	***P*-value**
Number	8	2.34	239	69.88	95	27.78	—
Age,year							
≤60	2	4.35	37	80.43	7	15.22	0.204
>60	11	3.72	198	66.89	87	29.39	
Gleason score							<0.001
Gleason 2-6	5	15.15	21	63.64	7	21.21	
Gleason 7	3	2.29	109	83.21	19	14.50	
Gleason 8-10	4	5.20	22	28.57	51	66.23	
PC							
CRPC	4	3.92	15	14.71	83	81.37	<0.0005
HDPC	5	3.60	119	85.61	15	10.79	
HGPIN and Nor							
BPH	0	0	19	95.00	1	5.00	0.970
HGPIN	1	1.64	57	93.44	3	4.92	
Normal tissue	1	5.00	18	90.00	1	5.00	

As shown in Table 1, 18 (90%) out of 20 cases of normal prostate tissue, 19 (95%) out of 20 cases of BPH, and 56 (92%) out of 61 cases of HGPIN showed weak (1+) expression of GOLPH3. There were no statistically significant differences in GOLPH3 expression of BPH, normal prostate tissues, or HGPIN. 83 (81.37%) of 102 CRPC cases showed moderate/intense expression for GOLPH3, whereas 15 (14.71%) cases were detected with weak expression. On the other hand, 4 (3.92%) cases showed negative results for GOLPH3 expression. 15 (10.79%) of 139 HDPC cases showed moderate/intense expression of GOLPH3, while 119 (85.61%) reported weak expression. In contrast, 5 (3.60%) cases were tested negative for GOLPH3 expression. Moreover, moderate/intense expression of GOLPH3 was found in 3 (4.92%) of 61 HGPIN, 1 (5%) of 20 BPH cases, and 1 (5%) of 20 normal prostate tissue. There were statistically significant differences in GOLPH3 expression (moderate/intense) of CRPC and HDPC (*P* < 0.0005). In contrast, there was no statistical difference in GOLPH3 expression (moderate/intense) of HDPC and HGPIN, BPH, and normal prostate tissue. Moderate/intense expression of GOLPH3 was reported in CRPC cases (*P* = 0.012). As shown in Table 2, CRPC cases also reported higher Gleason score (*P* = 0.017), bone metastasis (*P* = 0.024), higher baseline PSA (*P* = 0.038), and higher PSA nadir (*P* = 0.032) .

**Table 2:** Correlationship between clinicopathologic characteristics of patients and intensity of GOLPH3 expression

**Characteristics**	**Intensity of GOLPH3 expression**	
	***P *value**	***C *value**
Androgen dependence	0.012	0.144
Yes		
No		
Gleasone Score	0.017	0.421
Gleason 2-6		
Gleason 7		
Gleason 8-10		
Bone metastasis	0.024	0.398
Yes		
No		
Baseline PSA	0.038	0.181
≤10ng/ml		
>10ng/ml		
PSA nadir	0.032	0.312
≤1ng/ml		
>1ng/ml		

**Figure 2:**
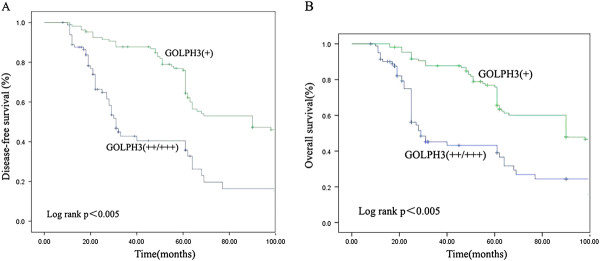
**Disease-free survival (A) and overall survival (B) curves according to the intensity of GOLPH3 expression**.

According to statistical research reports of June 2010, the average follow-up time was 38.5 months (range, 10–91 months). Among the 241 patients with prostate cancer, 44 (18.26%) were lost to follow-up and 31 (12.86%) succumbed to death. In the five-year period, the DFS and OS rates were 68.2 and 71.6%, respectively. All the 241 prostate cancer patients were examined to determine their GOLPH3 status. In the five-year period, the DFS rates of patients with moderate/intense GOLPH3 expression was 43.1%. On the other hand, DFS rates for patients with weak GOLPH3 expression was 87.2% during the five-year study period. The overall survival rate of patients with moderate/intense GOLPH3 expression was 45.4%. But, the overall survival rate of patients with weak GOLPH3 expression was 89.3%. Compared to patients with moderate/intense GOLPH3 expression, patients with weak GOLPH3 expression had a significant longer DFS (HR = 0.28, *P* = 0.012) and OS (HR = 0.42, *P* = 0.027; data not shown) (Figure 2).

Table 3 enlists the statistically significant predictors of DFS within the univariate analysis. Higher Gleason score, higher prostate-specific antigen nadir, positive bone metastasis, and moderate/intense positive GOLPH3 expression were the parameters that were correlated with shorter DFS. Compared to patients with intense or moderate GOLPH3 expression, the patients whose tumor cells showed weak expression of GOLPH3 had significantly better outcomes in terms of DFS (*P*<0.001). In the multivariate analysis, moderate/intense GOLPH3 expression continued to be a significant predictor of DFS while simulating a model containing all the clinicopathologic variables (*P* = 0.027; Table 3).

**Table 3:** Univariate and multivariate analyses of disease-free survival in prostate patients

**Variables**	**Univariate**			**Multivariate**		
	**HR**	**95%CI**	***P *value**	**HR**	**95%CI**	***P *value**
Age,year (≤60 vs>60)	0.87	0.51―1.46	0.238	0.81	0.46―1.61	0.312
Gleasone Score (2–6 vs 7 or 8–10)	4.37	2.64―5.81	<0.001	5.04	3.61―6.47	<0.001
Bone metastasis (Yes vs No)	5.56	3.67―7.02	<0.001	5.44	3.46―7.11	<0.001
Baseline PSA (≤10 vs>10ng/ml)	3.24	1.89―4.51	<0.001	3.03	1.94―5.74	<0.001
PSA nadir (≤1 vs 1ng/ml)	4.21	2.97―5.84	<0.001	4.76	3.43―6.56	<0.001
Androgen dependence (Yes vs No)	0.64	0.37―1.53	<0.001	0.71	0.44―1.49	<0.001
GOLPH3 (++/+++ vs +)	0.45	0.34―0.69	0.004	0.54	0.41―0.69	0.027

## Discussion

Prostate cancer is a serious illness that continues to present several different challenges to the urologists, pathologists, radiologists and oncologists [11]. In recent times, the incidence rate of prostate cancer has been steadily increasing in China. Prostate cancer is the most common non-dermatologic cancer. Despite the high incidence rate of prostate cancer, its clinical course of treatment is often unpredictable [12,13]. While treating prostate cancer at an advanced stage, patients are subjected to surgical or pharmacological castration. This is the most widely accepted method of treating prostate cancer at an advanced stage. However, the prostate tumor becomes hormone refractory after a period of 14 to 36 months. As the prostate tumor undergoes a transition from the hormone refractory stage to metastatic stage, it poses severe problems in clinical management [14]. Gleason score [15], PSA [16,17], clinical stage, and prostate volume are crucial parameters that need to be considered in the treatment of prostate cancer. Gleason score is an important prognostic factor for predicting biochemical failure, systemic recurrence, and overall patient survival [18]. However, there may be variations in comprehending the Gleason score among pathologists [19]. Note that, elevated levels of PSA can also be associated with BPH and prostatitis [20]. Therefore, these biomarkers seem inadequate to precisely determine the possibility of recurrence or metastasis. Therefore, the underlying molecular mechanisms of prostate carcinogenesis should be further investigated and elucidated. These efforts will help us in finding a reliable biomarker that can help us in improving the evaluation and prognosis of patients with CRPC [21].

In this study, GOLPH3 expression was detected in most benign and malignant tissues. It is interesting to note that 98 (40.66%) of 241 prostate cancer showed GOLPH3 moderate/intense expression. This result confirmed the findings of previous studies that reported an over expression of GOLPH3 in 37% of prostate cancer cases [7].

Progression to androgen-independence is a complex process involving various combinations of clonal selection [22], adaptive up-regulation of anti-apoptotic genes [23-25], androgen receptor transactivation in the absence of androgen or increased levels of coactivators [26,27], and alternative growth factor pathways [27,28]. According to this study, no statistically significant difference was found in GOLPH3 expression of normal prostate tissue, BPH, and HDPC. In contrast, there was a statistical difference in GOLPH3 expression of CRPC and HDPC cases (p<0.0005). These findings suggested that the oncogene GOLPH3 was highly conserved throughout evolution. In fact, GOLPH3 was strictly regulated in normal tissues, since it was essential for normal cell growth. Furthermore, these data suggest the possibility of associating the over-expression of GOLPH3 with the progression of prostate cancer. This probability is more pronounced in the transition from hormone-sensitive to hormone-refractory tumors. But, we cannot decipher the correlation of GOLPH3 expression with cellular hyperproliferation and tumorigenesis, especially during the early stages of prostate cancer development.

mTOR is a serine/threonine protein kinase, which is found in both rapamycin-sensitive and rapamycin-insensitive multimeric protein complexes. To regulate cell growth, cell-cycle progression, and cell differentiation, mTOR functions as a central signal integrator that receives signals from growth factors, nutrients, and cellular energy metabolism [29,30]. Therefore, mTOR is recognized as a central coordinator of these fundamental biological processes [31-33]. Note that, mTOR is an evolutionarily conserved protein kinase. Recent studies have reproted that GOLPH3 works as an oncoprotein promoting cell transformation and tumor growth by enhancing the activity of mTOR [7]. Since mTOR is required for cell differentiation, hyperactivation of mTOR may be associated with abnormal cell differentiation. In conclusion, an overexpression of GOLPH3 causes an abnormal differentiation of prostate cancer cells, thereby creating heterogeneity of tumor cells. New subclones with altered growth properties proliferate owing to this trait of heterogeneity. In fact, the transition from hormone-sensitive to hormone-refractory tumors can be probably attributed to this molecular mechanism.

In this research study, it was found that the incidence of Gleason score, PSA nadir, baseline PSA, and positive bone metastasis was higher in patients detected with moderate/intense GOLPH3 expression. In our study, we also demonstrated that GOLPH3 over-expression was significantly associated with a shorter DFS and OS. Multivariate analysis revealed a significant negative relationship between the over expression of GOLPH3 and DFS or OS. Therefore, we can conclude that GOLPH3 serves as a biomarker for predicting the severity of prostate cancer. GOLPH3 expression is an important parameter used in the prognosis of prostate cancer patients.

In this research study, we have discovered that GOLPH3 expression does not have any correlation with cellular hyperproliferation and tumorigenesis, particularly in the early stages of prostate cancer. On the other hand, over-expression of GOLPH3 could be correlated with the progression of prostate cancer from its hormone sensitive phase to hormone refractory phase. Furthermore, GOLPH3 might be a favorable prognostic factor of DFS and OS in patients diagnosed with prostate cancer. In this way, GOLPH3 expression serves as a reliable prognostic marker. In fact, determining the expression of GOLPH3 might also help in further elucidating the risk of progression of prostate cancer in patients. In conclusion, GOLPH3 can be a novel candidate for the development of an effective therapeutic strategy for CRPC.

## Competing interest

The authors declare no conflict of interest.

## Authors’ contributions

XH, LNY and HS participated in the design of the study. XH wrote the manuscript. XXH and QX carried out the H&E and IHC staining. WHP and LF collected the clinical data and reviewed H&E and IHC slides. ZXL performed the statistical analysis. All authors read and approved the final manuscript.

## References

[B1] Niu Z Guohua R Shuping S Diagnosis and treatment for prostate cancer. Chinese–German J Clin Oncol 2008 7 492 494

[B2] Debes JD Tindall DJ Mechanisms of androgen-refractory prostate cancer N Engl J Med 2004 351 1488 1490 10.1056/NEJMp048178 15470210

[B3] Fritzsche FR Stephan C Gerhardt J Lein M Hofmann I Jung K Dietel M Kristiansen G Diagnostic and prognostic value of T-cell receptor gamma alternative reading frame protein (TARP) expression in prostate cancer Histol Histopathol 2010 25 733 739 20376779 10.14670/HH-25.733

[B4] Parekh DJ Ankerst DP Troyer D Srivastava S Thompson LM Biomarkers for prostate cancer detection J Urol 2007 178 2252 2259 10.1016/j.juro.2007.08.055 17936845

[B5] Dippold HC Ng MM Farber-Katz SE GOLPH3 bridges phosphatidylinositol-4-phosphate and actomyosin to stretch and shape the Golgi to promote budding Cell 2009 139 337 351 10.1016/j.cell.2009.07.052 19837035 PMC2779841

[B6] Wood CS Schmitz KR Bessman NJ PtdIns4P recognition by Vps74/GOLPH3 links PtdIns 4-kinase signaling to retrograde Golgi trafficking J Cell Biol 2009 187 967 975 10.1083/jcb.200909063 20026658 PMC2806290

[B7] Scott KL Kabbarah O Liang MC Ivanova E Anagnostou V Wu J Dhaka S Wu M Chen SJ Feinberg T Huang J Saci A Widlund HR Fisher DE Xiao YH Rimm DL Protopopov A Wong KK Chin L GOLPH3 modulates mTOR signalling and rapamycin sensitivity in cancer Nature 2009 459 1085 1090 10.1038/nature08109 19553991 PMC2753613

[B8] Abraham RT GOLPH3 links the Golgi network to mTOR signaling and human cancer Pigment Cell Melanoma Res 2009 22 378 379 10.1111/j.1755-148X.2009.00596.x 19624311

[B9] Kononen J Bubendorf L Kallioniemi A Barlund M Schraml P Leighton S Torhorst J Mihatsch MJ Sauter G Kallionimeni OP Tissue microarrays for high-throughput molecular profiling of tumor specimens Nat Med 1998 4 844 847 10.1038/nm0798-844 9662379

[B10] He J Peng R Yuan Z Wang S Peng J Lin G Jiang X Qin T Prognostic value of androgen receptor expression in operable triple-negative breast cancer: a retrospective analysis based on a tissue microarray Med Oncol 2012 29 406 410 10.1007/s12032-011-9832-0 21264529

[B11] Jemal A Siegel R Ward E Murray T Xu J Smigal C Thun MJ Cancer statistics, 2006 CA Cancer J Clin 2006 56 106 130 10.3322/canjclin.56.2.106 16514137

[B12] Levi M Cancer and DIC Haemostasis 2001 31 47 48 11990477

[B13] de la Fouchardière C Flechon A Droz JP Coagulopathy in prostate cancer Neth J Med 2003 61 347 354 14768717

[B14] Wedel S Hudak L Seibel J-M Makarevic J Juengel E Tsaur I Waaga-Gasser A Haferkamp A Blaheta RA Molecular targeting of prostate cancer cells by a triple drug combination down-regulates integrin driven adhesion processes, delays cell cycle progression and interferes with the cdk-cyclin axis BMC Cancer 2011 11 375 10.1186/1471-2407-11-375 21867506 PMC3170298

[B15] Moussa AS Li J Soriano M Klein EA Dong F Jones JS Prostate biopsy clinical and pathological variables that predict significant grading changes in patients with intermediate and high grade prostate cancer BJU Int 2009 103 43 48 10.1111/j.1464-410X.2008.08059.x 18782303

[B16] Denham JW Lamb DS Joseph D Matthews J Atkinson C Spry NA Duchesne G Ebert M Steigler A Este CD PSA response signatures – a powerful new prognostic indicator after radiation for prostate cancer? Radiother Oncol 2009 90 382 388 10.1016/j.radonc.2008.10.004 18992951

[B17] Kelloff GJ Coffey DS Chabner BA Dicker AP Guyton KZ Nisen PD Soule HR Amico AVD Prostate-Specific Antigen Doubling Time as a Surrogate Marker for Evaluation of Oncologic Drugs to Treat Prostate Cancer Clin Cancer Res 2004 10 3927 3933 10.1158/1078-0432.CCR-03-0788 15173102

[B18] Tollefson MK Leibovich BC Slezak JM Zincke H Blute M Long-term prognostic significance of primary Gleason pattern in patients with Gleason score 7 prostate cancer: Impact on prostate cancer specific survival J Urol 2006 175 547 551 10.1016/S0022-5347(05)00152-7 16406993

[B19] Helpap B Egevad L Modified Gleason grading. An updated review Histol Histopathol 2009 24 661 666 19283673 10.14670/HH-24.661

[B20] Hara N Kitamura Y Saito T Komatsubara S Total and free prostate-specific antigen indexes in prostate cancer screening: value and limitation for Japanese populations Asian J Androl 2006 8 429 434 10.1111/j.1745-7262.2006.00155.x 16763718

[B21] Bin W He W Feng Z Xiangdong L Yong C Lete K Hongbin Z Honglin G Prognostic relevance of cyclooxygenase-2 (COX-2) expression in Chinese patients with prostate cancer Acta Histochem 2011 113 131 136 10.1016/j.acthis.2009.09.004 19836060

[B22] Isaacs JT Wake N Coffey DS Sandberg AA Genetic instability coupled to clonal selection as a mechanism for tumor progression in the Dunning R-3327 rat prostatic adenocarcinoma system Cancer Res 1982 42 2353 2371 7074614

[B23] Miyake H Tolcher A Gleave ME Antisense Bcl-2 oligodeoxynucleotides inhibit progression to androgenindependence after castration in the Shionogi tumor model Cancer Res 1999 59 4030 4034 10463603

[B24] Miyake H Nelson C Rennie PS Gleave ME Testosterone-repressed prostate message-2 is an antiapoptotic gene involved in progression to androgen independence in prostate cancer Cancer Res 2000 60 170 176 10646870

[B25] Miyake H Pollak M Gleave ME Castration-induced upregulation of insulin-like growth factor binding protein-5 potentiates insulin-like growth factor-I activity and accelerates progression to androgen-independence in prostate cancer models Cancer Res 2000 60 3058 3064 10850457

[B26] Bubulya A Wise SC Shen XQ Burmeister LA Shemshedini L c-Jun can mediate androgen receptorinduced transactivation J Biol Chem 1996 271 24583 24589 10.1074/jbc.271.40.24583 8798722

[B27] Sato N Sadar MD Bruchovsky N Saatcioglu F Rennie PS Sato S Lange PH Gleave ME Androgenic induction of prostate-specific antigen gene is repressed by protein–protein interaction between the androgen receptor and AP-1/c-Jun in the human prostate cancer cell line LNCaP J Biol Chem 1997 272 17485 17494 10.1074/jbc.272.28.17485 9211894

[B28] Craft N Shostak Y Carey M Sawyers CL A mechanism for hormone-independent prostate cancerthrough modulation of androgen receptor signalingby the HER–2/neu tyrosine kinase Nat Med 1999 5 280 285 10.1038/6495 10086382

[B29] Fingar DC Salama S Tsou C Harlow E Blenis J Mammalian cell size is controlled by mTOR and its downstream targets S6K1 and 4EBP1/eIF4E Genes Dev 2002 16 1472 1487 10.1101/gad.995802 12080086 PMC186342

[B30] Feng Z Zhang H Levine AJ Jin S The coordinate regulation of the p53 and mTOR pathways in cells Proc Natl Acad Sci USA 2005 102 8204 8209 10.1073/pnas.0502857102 15928081 PMC1142118

[B31] Jacinto E Hall MN Tor signalling in bugs, brain and brawn Nat Rev Mol Cell Biol 2003 4 117 126 10.1038/nrm1018 12563289

[B32] Oldham S Hafen E Insulin/IGF and target of rapamycin signaling: a TOR de force in growth control Trends Cell Biol 2003 13 79 85 10.1016/S0962-8924(02)00042-9 12559758

[B33] Schmelzle T Hall MN TOR, a central controller of cell growth Cell 2000 103 253 262 10.1016/S0092-8674(00)00117-3 11057898

